# Preparation, Characterization, and Evaluation of Cytotoxicity of Fast Dissolving Hydrogel Based Oral Thin Films Containing Pregabalin and Methylcobalamin

**DOI:** 10.3390/gels9020147

**Published:** 2023-02-09

**Authors:** Emrah Özakar, Rukiye Sevinç-Özakar, Bilal Yılmaz

**Affiliations:** 1 Department of Pharmaceutical Technology, Faculty of Pharmacy, Atatürk University, 25240 Erzurum, Turkey; 2 Department of Analytical Chemistry, Faculty of Pharmacy, Atatürk University, 25240 Erzurum, Turkey

**Keywords:** dysphagia, geriatric, pediatric, oral thin film, OTF, pregabalin, methylcobalamin, neuropathic pain, solvent pouring method, cytotoxicity

## Abstract

The oral availability of many drugs is problematic due to the pH of the stomach, enzymes, and first-pass effects through the liver. However, especially geriatric, pediatric, bedridden, or mentally handicapped patients and those with dysphagia have difficulty swallowing or chewing solid dosage forms. Oral Thin Films (OTFs) are one of the new drug delivery systems that can solve these problems. Pregabalin (PG) and Methylcobalamin (MC), which are frequently preferred for pain originating in the central nervous system, were brought together for the first time using OTF technology in this study. In this study, a quantification method for PG and MC was developed and validated simultaneously. Optimum formulations were selected with organoleptic and morphological controls, moisture absorption capacity, swelling capacity, percent elongation, foldability, pH, weight variability, thickness, disintegration time, and transparency tests on OTFs prepared by the solvent pouring method. Content uniformity, dissolution rate, determination of release kinetics, SEM, XRD, FT-IR, DSC, long-term stability, and cytotoxicity studies on the tongue epithelial cell line (SCC-9) were performed on selected OTFs. As a result, OTFs containing PG-MC, which are non-toxic, highly flexible, transparent, compatible with intraoral pH, with fast disintegration time (<30 s), and acceptable in taste and appearance, have been developed successfully.

## 1. Introduction

The oral availability of many drugs is inadequate due to gastric pH, enzymes, and extensive first-pass effects through the liver. These drugs have to date been traditionally administered parenterally and have demonstrated low patient compliance. However, some patient groups, especially pediatric and geriatric patients, have difficulty swallowing or chewing solid dosage forms. Many pediatric and geriatric patients are reluctant to take these solid dosage forms for fear of choking. This situation has paved the way for the pharmaceutical industry to develop alternative systems for the transport of drugs by developing orally disintegrating/soluble thin films [1,2,3]. Orally disintegrating/soluble thin films or strips are defined as “drug delivery systems containing a water-soluble polymer that, when placed on the tongue or in the oral cavity, quickly disperses with saliva, dissolves or adheres to the mucous membrane, and releasing the drug within a few seconds” [4,5]. OTFs have a rising market portion today due to their dosage form, ease of administration, and high efficacy [6]. The OTF dosage form has started to be especially preferred in many prescription and over-the-counter preparations and multivitamin combinations [7]. OTFs can be prepared in a variety of ways. The most commonly used and preferred method is the Solvent Pouring Method. In this method, which is also used within the scope of our study, the water-soluble components that make up the OTF formulation are mixed by heating. Afterward, the active substance and other excipients are added to this mixture to obtain a viscous solution. This solution is poured into a petri dish, and the solvents are allowed to evaporate. The advantages of the solvent pouring method include the fact that OTFs are clear and bright, highly flexible, have excellent and thin film thickness uniformity, and are cost-effective [1,8,9].

The International Association for the Study of Pain (IASP) defines neuropathic pain (NP) as “pain initiated or caused by a primary lesion or dysfunction in the nervous system.” NP can be controlled with various drugs, including nonsteroidal anti-inflammatory drugs, local anesthetics, and narcotic analgesics. In a study, it was reported that nonsteroidal analgesics for NP were generally insufficient for treatment, whereas the Pregabalin–Vitamin B12 combination gave safe, effective, and well-tolerated results with minor side effects [10].

**Figure 1:**
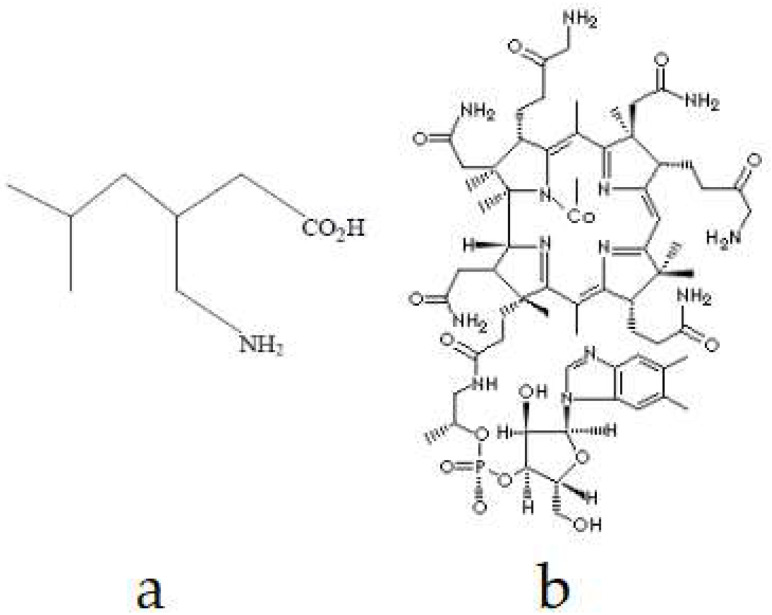
Chemical structure of PG (**a**) [16,17] and MC (**b**) [18].

PG is an anticonvulsant and analgesic drug. Its mechanism of action has only been partially characterized. In particular, the cellular and molecular details of the effect of reducing neurotransmitter release are not fully known [11,12]. The tmax duration of PG, which is classified as a BCS Class I drug, is around 1.5–3 h. It has more than 90% bioavailability and PG absorption is dose-independent (Figure 1) [13]. PG, a member of the gamma aminobutyric acid class, is considered one of the first drugs to come to mind for the treatment of NP. PG has been proven to be effective for both central and peripheral NP and provides rapid pain relief [14]. It does not bind directly to GABAa or GABAb receptors; GABA shows its effect by increasing the density of carrier proteins [13].

MC (Vitamin B12) [15,16] is a water-soluble B group vitamin obtained from the dietary intake of animal proteins, mainly meat and eggs (Figure 1). MC plays an important role in the production of red blood cells, DNA synthesis, and the regulation of neurological functions. MC deficiency causes the demyelination of nerves in the central and peripheral nervous system, which is associated with peripheral neuropathy, Alzheimer’s, loss of sensation in peripheral nerves, and weakness in the lower extremities [12]. MC has an important role in regenerating the myelin sheath and helps restore the function of the nerve in neuropathy [14].

In several clinical studies, fixed-dose combinations of PG and MC have been administered to patients to evaluate pain management in a population of patients with low to moderate painful diabetic neuropathy. In these studies performed with different dose combinations, it was reported that there was a statistically significant decrease in the neuropathic pain of the patients and that the tolerance of the patients was quite high. It has even been reported that the accompanying use of painkillers and muscle relaxants decreased to less than 1/3 [19]. The combined use of PG and MC plays a major role in increasing patient tolerance and reducing the severity of NP.

The novelty of this study is that hydrogel-based thin film formulations will be the first in the literature, considering that the fixed-dose combination of PG and MC combined drug therapy, which is preferred for nerve pain (especially in NP), offers an effective and appropriate treatment with minimal side effects. In this study, it was aimed that dysphagic, geriatric, pediatric, bedridden, or mentally retarded patients with NP complaints such as diabetic neuropathy can easily use the combination as a thin film containing the two active substances together without experiencing swallowing problems and without the need for water. Our study aimed not only to design an alternative dosage form to conventional drug treatments but also to develop OTF formulations that provide a good mouth feel, acceptable organoleptic properties, and sufficiently flexible, thin, and appropriate tensile strength for patient compliance. As a result of the absorption of OTFs from the sublingual absorption region, the first-pass effect through the liver is eliminated, and the dissolution of the thin films in the sublingual and/or intraoral application, and especially from the sublingual region, is very rapid. It is also possible to get a quick response to nerve pain. In this way, a significant advantage will be gained over treatments with conventional drugs (such as conventional tablets and capsules).

Within the scope of our study, many OTF formulation development studies were carried out with combinations of active substances, film-forming polymers, and other excipients. Various characterization studies have been carried out to determine the optimum formulations. These studies include compatibility (FT-IR), organoleptic and morphological controls, moisture sorption capacity, swelling capacity, percent elongation, pliability (flexibility), pH, weight variability, thickness, disintegration time, and transparency. With the data obtained here, content uniformity, dissolution rate, determination of release kinetics, SEM, XRD, FT-IR, DSC, long-term stability, and cytotoxicity studies on the tongue epithelial cell line (SCC-9) were also performed on the selected formulations [5,20,21].

## 2. Results and Discussion

### 2.1. Quantification Method Development and Validation of the Pregabalin and Methylcobalamin

**Figure 2:**
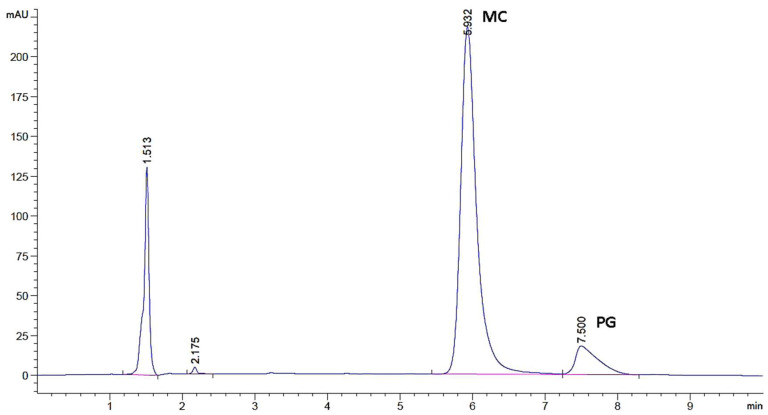
Chromatogram of PG and MC.

The quantification method was developed and validated by making modifications on the simultaneous determination methods of PG and MC in the literature. The HPLC chromatogram and chromatographic conditions are given in Table 1 and Figure 2. The calibration equations were found y = 595.02x − 5319.9 (R^2^ = 0.9971 for PG) and y = 249284x + 9918.5 (R^2^ = 0.9991 for MC).

#### 2.1.1. Linearity

Concentrations within the calibration curve (for PG: 10, 25, 50, 100, 200, 300, 400, 500 µg/mL; for MC: 0.25, 0.5, 1, 2.5, 5, 10, 25, 50, 100 µg/mL) and the intervals in which the method is linear were determined.

#### 2.1.2. Accuracy and Precision

Three points in the calibration curve (for PG: 37.5, 150, 450 µg/mL, for MC: 1.25, 12.5, 75 µg/mL) were determined; analyses were made for three consecutive days, intraday and interday. The relevance of the intraday and interday results for three consecutive days to the theoretical values was calculated statistically. The results are given in Table 2.

#### 2.1.3. LOD and LOQ

Experimentally, was determined that our LOD value is 0.075 µg/mL for MC and 3 µg/mL for PG. Our LOQ value was found to be 0.25 µg/mL for MC and 10 µg/mL for PG.

#### 2.1.4. Selectivity (Specificity)

With the HPLC quantification method, PG and MC were analyzed in the presence of components forming OTF formulations, and the originality of the method was proven.

#### 2.1.5. Stability of Active Substances

Pure PG and MC were kept in solution at room temperature for 72 h and their quantifications were made using a validated method. As a result of this period, it was determined that their recovery was within the limits of 95–105% quality control and they were stable (Table 3).

### 2.2. Preparation and Organoleptic Evaluation of OTFs

**Figure 3:**
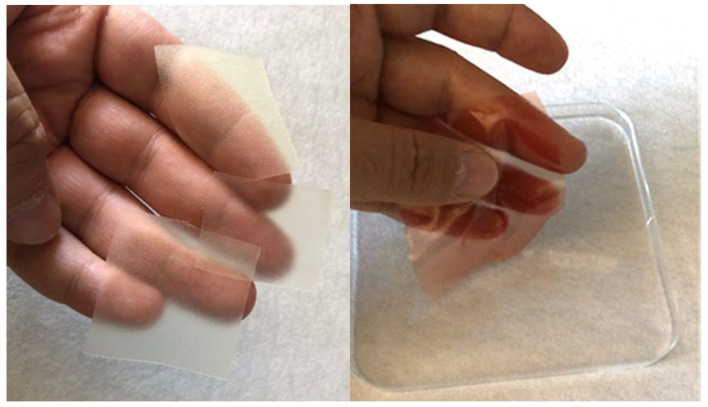
Blank OTFs (**left**), PG and MC included OTF (**right**).

OTFs containing active substances and blank OTFs have been developed successfully. In Figure 3, the images are given of the film samples containing blank and active substances. The organoleptic evaluation data of the OTF formulations are given in Table 4.

In the evaluation by Hoffman et al., it was stated that an ideal film thickness to be used orally should be between 12 and 100 μm [22]. Film thickness is a very important parameter in terms of homogeneity. It directly affects the correct dosage and release properties of the film. The films are expected to be thin to ensure rapid dissolution when exposed to water or saliva. Increasing the thickness will change the disintegration time and release kinetics. Transparency will also change with thickness. The duration of the drug in the mouth will vary in direct proportion to the thickness of the film. 

In other studies using “pullulan” as the film-forming polymer, as in our research, the film thickness was determined by Panchal et al., 70 µm [23]; determined by Sharma et al., 100 µm [24]; and determined by Pezik et al., 38–70 µm [25]. It was observed that with a decrease in the film thickness, the polymer degradation accelerated, and thus the release kinetics changed. As seen in Figure 3, the OTF samples appear very homogeneous, thin and transparent. In addition, all OTF thicknesses were found to be 30 µm.

### 2.3. Moisture Absorption Capacity

The moisture absorption capacities of the formulations were examined (Table 5), and it was determined that the formulations generally did not have moisture absorption properties (<2%). The moisture absorption capacity was statistically different in OTFs prepared using NaCMC, pullulan, pectin, and sodium alginate from OTFs prepared with other polymers (*p* < 0.05). This may be due to the fact that the polymers used have the property of entrapping water in the engagement of their structure. Depending on the hydrogel formation, water may be retained in the structure, and the environment’s moisture may be entrapped in the polymeric structure.

### 2.4. Tensile Strength (Elongation)

**Figure 4:**
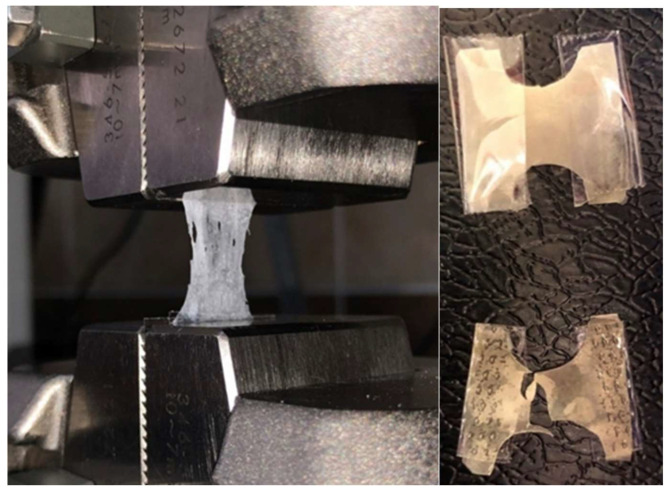
Elongation percentage experiment and images taken during the experiment.

Flexibility directly affects the structural integrity and stability of the films. In order to determine this, a force was applied to extend each OTF formulation by 1 mm per minute. Sample images from the measurements are given in Figure 4, and the data on the elongation percentages of the formulations are given in Table 5.

The tensile strength of the prepared OTF formulations were examined, and it was seen that the polymers used in the formulations, and especially the plasticizers, had a direct effect. It was determined that formulations using glycerin as a plasticizer were more flexible (F4, F5, F6, F11, F12, F13, F14, F15, F16, and F17). In particular, it was observed that the formulations using propylene glycol had no elongation capacity at all (F1, F2, F3, F7, F9, F10, and F18). It was determined that the elongation capacity increased when PEGs were added to the formulations as a plasticizer (F19, F20, F23, and F24). It was observed that low molecular weight PEGs (PEG 400) increased the elongation capacity (F19 and F23), while high molecular weight PEGs (PEG 4000) lowered the elongation capacity (F20 and F24). Statistically, it was determined that OTF formulations prepared using the plasticizer glycerin provided significant flexibility compared to OTFs prepared using propylene glycol, PVA, and D-sorbitol, indicating a significant difference in their elongation capacity (*p* < 0.05).

### 2.5. Weight Variability and Thickness

The weight variations of the OTF formulations are given in Table 5. The results were examined, and it was determined that all formulations had almost the same thickness, with no statistically significant difference (*p* < 0.05).

### 2.6. Folding Endurance (Flexibility)

The folding endurance of OTFs is an extremely important parameter in terms of the stability of thin films and their ease of use by patients until the last moment of their shelf-life. The data of the tests for the folding endurance of the formulations are given in Table 5. It was determined that formulations using glycerin as the plasticizer could be folded several hundred times (F4, F5, F6, F11, F12, F13, F14, F15, F16, and F17). Meanwhile, formulations using propylene glycol were found to be the least foldable (F1, F2, F3, F7, F9, F10, and F18). This difference was statistically significant compared to the formulation prepared with glycerin (*p* < 0.05).

### 2.7. Determination of pH Values

The pH of OTFs means that, when placed on the tip of the tongue, they should be able to dissolve or disperse by saliva contact in accordance with the intraoral pH. That’s why pH is important. The pH of each OTF formulation was measured and is given in Table 5. Citric acid added to formulations is a saliva stimulant. Since the pH of formulations is near or slightly lower than the intraoral pH value, it will result in more saliva secretion, and the formulations’ disintegration/dissolution/dispersion will be accelerated. The pHs of the formulations were found in the range of 5.11 to 6.55.

### 2.8. Determination of Swelling Degrees

The swelling of OTFs with saliva in the oral media and their water absorption capacity is a desirable condition. However, excessive water intake and swelling also prolong the residence time of the formulation in the mouth and creates an unpleasant effect in terms of patient compliance. With the measurements, the percent swelling capacities of each OTF were determined, and the results are given in Table 5. The results were examined, and it was determined that the F5, F8, and F13 formulations exhibited the least degree of swelling; this difference was statistically significant compared to other OTF formulations (*p* < 0.05). It was observed that the formulations prepared with pullulan exhibited less swelling capacity, but the water absorption capacity increased with the addition of sodium alginate or cellulose derivatives to the structure. Here, pullulan and sodium alginate were found to be important choices in the design of OTFs.

### 2.9. Disintegration Test

In OTFs, the disintegration and dissolution rate processes are difficult to distinguish because of the short time in which they occur. OTFs are currently not registered with any pharmacopoeia. For this reason, for OTFs, the International Pharmaceutical Federation/American Association of Pharmaceutical Scientists (FIP/AAPS) has stated that the disintegration test can be used instead of the dissolution rate test as in ODTs. While the European Pharmacopoeia has given a disintegration time of up to 3 min for ODTs, this time is recommended as 30 s or less according to FDA (American Food and Drug Administration) and USP (American Pharmacopoeia) guidelines. Since the volume of saliva in the oral cavity is less than 2 mL, these tests are generally recommended for disintegration testing in a small environment in 2–7 mL of liquid under conditions similar to those in the oral cavity [26].

The disintegration time of OTFs in the oral cavity is one of the important parameters in the design of OTFs. For this purpose, the data of this test performed for each OTF formulation are given in Table 5.

The critical component in formulations for the dispersion/disintegration of OTFs is superdisintegrants. For this purpose, sodium starch glycolate, xanthan gum, PVP-CL, and croscarmellose sodium were used in the formulations. The disintegration times were examined in formulations prepared with pullulan, sodium alginate, or pectin. It was determined that the formulations using croscarmellose sodium as superdisintegrants disintegrated in a shorter time (F5, F7, F8, F13, F19, and F20). The OTFs prepared with the same polymers (pullulan, sodium alginate, or pectin) were found to disperse later in formulations using sodium starch glycolate as a superdisintegrant compared to in formulations using croscarmellose sodium, which was statistically significant (*p* < 0.05).

### 2.10. Transparency

The transparency of OTFs is important in terms of patient compliance and preferability. Transparency analysis was performed for each OTF formulation; the results are given in Table 5. The transparency of OTF formulations (such as F1, F6, F7, F11, and F12) prepared alone or in a combination of cellulose derivatives (such as sodium carboxymethyl cellulose, hydroxy propylmethylcellulose (HPMC E15) and pullulan as polymers were found to be significantly different compared to ones prepared with other polymers (*p* < 0.05). At the same time, it was found statistically significant (*p* < 0.05) that the transparency of OTFs prepared using croscarmellose sodium as a superdisintegrant agent was lower than that of the OTF formulations prepared using sodium starch glycolate or xanthan gum (such as F6, F7, and F8). In addition, propylene glycol, which is used as a plasticizer in formulations, affected the transparency of the formulations and contributed to their opaque appearance. This situation showed that there was a significant difference (F9 and F10 and F11 and F12) compared to formulations using glycerin as a plasticizer (*p* < 0.05).

The organoleptic controls (taste, odor, texture, transparency, thickness, flexibility, and homogeneity) of 24 developed OTF formulations, as a result of preformulation studies using the Solvent Casting Method, have been given in Table 4. All formulations were subsequently performed to the other tests; analyses are given in Table 5. These data were evaluated, and three OTF formulations (F5, F8 and F13) with optimum properties were selected for use in all other analysis and characterization studies.

### 2.11. Content Uniformity

The amount of each active substance was determined by the validated HPLC method from the F5, F8, and F13 formulations with optimum properties from the developed OTFs. The content uniformity results of PG and MC obtained are given in Table 6 for each formulation.

### 2.12. Dissolution Rate Test

Drug release from OTFs has usually been accomplished according to pharmacopoeial requirements for solid oral dosage forms in an environment adjusted at 37 °C (pH 6.8 phosphate buffer or artificial salivary fluid), using a basket or paddle apparatus. However, the dissolution device has some disadvantages for OTFs. Using the basket apparatus may result in the film sticking to the edges and clogging of the basket pores, whereas with the use of the paddle apparatus OTFs are likely to float in the dissolution medium or localize to the bottom of the vessel, making data reproducibility difficult. Sinkers and double-sided tapes are used to prevent floating and imitate in vivo adhesion. Each film is fixed to a rectangular glass plate and placed on the bottom of the dissolution medium. As a result of rapid disintegration, the release of the active substances occurs very quickly, and samples of the analyzed fluid are taken in a short time [26]. One of the most important parameters for predicting the in vivo behavior of active substances is the rate and time of exit of the active substances from the dosage form [25]. The average dissolution rate test results are shown for three different optimized film formulations (F5, F8, and F13) in Table 7. From all formulations, approximately 75% of PG was released within 2.5 min, and more than 90% of MC was released within 2.5 min. All dosage forms gave all of both PG and MC to the dissolution medium at 5 min, and therefore all of the films were dissolved/disintegrated. The results obtained in this short time are in full agreement considering the duration time of OTFs in the mouth. The increase in the solubility of the active substances may be due to their amorphous structure presence in the film matrix. XRD and DSC analyses confirmed that both active substances were amorphous.

### 2.13. Determination of Release Kinetics

The release kinetics and release mechanisms of the OTFs were calculated using the “Microsoft Excel” program based on the dissolution rate test results of the optimum OTF formulations (F5, F8, and F13) containing PG-MC (Table 8). It was determined by mathematical calculations and formulas that the formulations would be compatible with the Zero Order, First Order, Higuchi, and Korsmeyer-Peppas models [27]. The mathematical modeling of the predictable consequences of drug release should be performed to better understand the release profiles of drugs and to predict their in vivo performance [28].

The predictability of the release kinetics of active substances represents a key feature applied to delivery systems for their acceptance as drugs. Both their safety and efficacy depend on the absorption of the active substances, their rate of reaching the site of action, and their amount. Measuring, estimating, and ultimately modeling release kinetics represents a complex investigation that requires an in-depth understanding of physicochemical, physiological, and mathematical processes [29].

The delivery of drugs from pharmaceutical preparations is associated with various physical and chemical parameters. It results in attributing appropriate mathematical models to the obtained release results. Zero Order kinetics can be used if the pharmaceutical dosage form does not disintegrate and slowly releases the drug. In the first-order kinetic model, the early dissolution stage of a poorly water-soluble drug embedded in a water-soluble matrix can be described. The Higuchi model is based on several assumptions. The initial concentration of the drug in the formulation is higher than the solubility of the drug; the drug only spreads in one dimension; the active substance particle is smaller than the size of a carrier; swelling and dissolution in the system are insignificant; drug diffusion does not change; sink conditions are achieved [30].

The R^2^ values were examined, and it was observed that all formulations conformed to Korsmeyer-Peppas release kinetics. The Korsmeyer-Peppas model is used when the drug release mechanism is not known exactly or when more than one type of release phenomenon is present. It is used to describe the release of this type of release drug from pharmaceutical dosage forms. All of our formulations exhibited release kinetics by this mechanism. The *n* value is important in formulations that comply with Korsmeyer-Peppas release kinetics. *n* = 0.45 indicates a Fickian diffusion mechanism; 0.45 < *n* < 0.89 indicates non-Fickian transport; *n* = 0.89 indicates case II (relaxational) transport; and *n* > 0.89 indicates Super Case II Transport. According to the release kinetic results of the formulations, “*n*” values were more than 0.45, and the release mechanism was seen as “Super Case II Transport” (*n* > 0.89). This indicates that all formulations are released by the “non-normal non-Fick” diffusion principle (Super Case II Transport mechanism). Non-Fickian drug release is controlled by a combination of diffusion and polymer relaxation. Here, it means that drug molecules diffuse through the highly hydrated polysaccharide matrix, which is involved in the dissolution or relaxation of polymer chains [31]. It can be said that, with the effect of the hydrogel matrix in the structure, rapid diffusion occurs with a quick liquid entry into the formulations. While there was no significant difference (*p* > 0.05) for PG release between the three formulations tested, F5 was found to be significantly different in MC release compared to the other two formulations (*p* < 0.05).

In a similar study in the literature, Rençber et al. prepared thin films with a Eudragit: HPMC polymer mixture and reported that the release kinetics followed the Korsmeyer-Peppas model and the release mechanism was non-Fickian (0.45 < *n* < 0.89) [31]. Zhang et al. reported that the release kinetics were in accordance with the Korsmeyer-Peppas model in their oral thin film studies prepared using cellulose derivatives. They found that the release mechanisms changed when they changed their film preparation method [32].

### 2.14. Fourier Transform Infrared (FT-IR) Analysis

**Figure 5:**
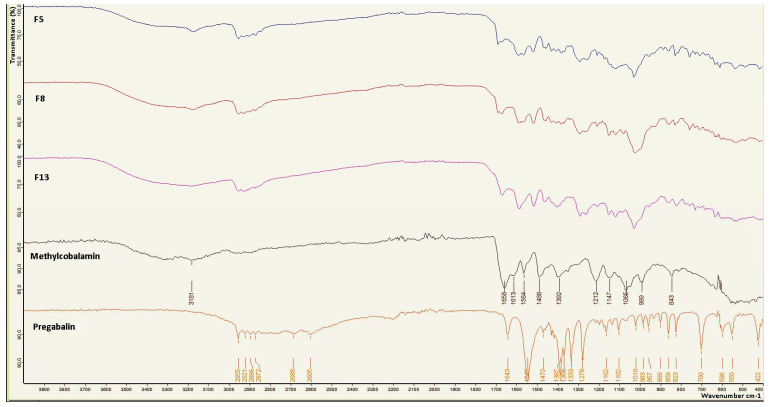
FT-IR spectra of PG, MC, and optimum formulations.

FT-IR spectroscopy analysis was performed on the prepared films to examine the interactions between active ingredients and excipients. Figure 5 shows the FT-IR spectra of the films. Pure PG showed peaks similar to those found in previous studies at 2955 cm^−1^ (C–H tension), 1643 cm^−1^ (K–H bending, NH_2_ shear), 1546 cm^−1^ (N–O asymmetric tension), 1470 cm^−1^ (C–H), 1333 cm^−1^ (N–O symmetrical stretch), 1278 cm^−1^ (C–O stretch), and 957 cm^−1^ (O–H bending) [33]. The FTIR spectrum of MC was examined, and it was seen that it showed a broad peak around 3400 cm^−1^ corresponding to the O–H group. Other peaks were observed in accordance with the literature at 1658, 1564, 1488, and 1065 cm^−1^ according to C=O, C=C, C=N, and PO_4_, respectively [34]. It was observed that some of the characteristic peaks of the active substances were suppressed in the formulation spectra of MC and PG. This situation leads to the conclusion that the drug remains in the polymeric matrix as a result of the drug/polymer complex, and the peaks may be suppressed for this reason. The characteristic peaks of PG and MC are also present in the formulations of hydrogel films. These peaks indicate that there is no drug–excipient interaction.

### 2.15. X-ray Diffraction (XRD) Analysis

**Figure 6:**
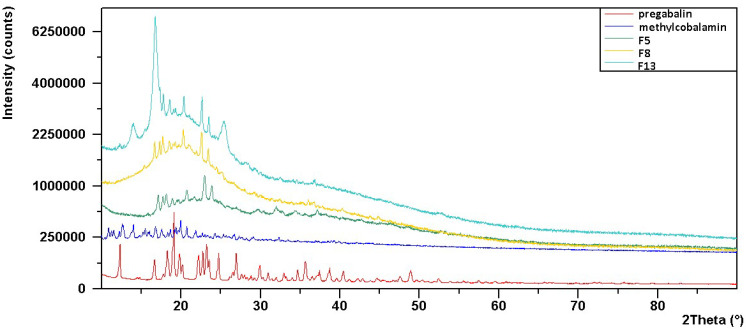
XRD diagrams of PG, MC, and optimum formulations.

The X-ray diffractogram of PG confirms its crystal structure, as evidenced by the number of sharp and intense peaks (Figure 6). The XRD pattern of pure PG shows characteristic peaks at 9.4, 19.04, 38.5, 40, and 49.9 [35]. It shows that the characteristic peaks of PG are in a partially amorphous state in all hydrogel-based OTFs. These results were consistent with those previously reported in the literature [35,36].

The X-ray diffractogram of MC confirms its crystal structure, as evidenced by the presence of sharp and intense peaks (Figure 6). For all three OTF formulations, there were three large peaks in the range of 20 to 25° and a less pronounced peak presence near 6° [37]. However, it was observed that the peaks representing the crystal structure of the active substances were suppressed in the diffractograms of the OTF formulations. The reason for this may be that the amorphous form becomes dominant in the formulation.

### 2.16. Differential Scanning Calorimetry (DSC) Analysis

**Figure 7:**
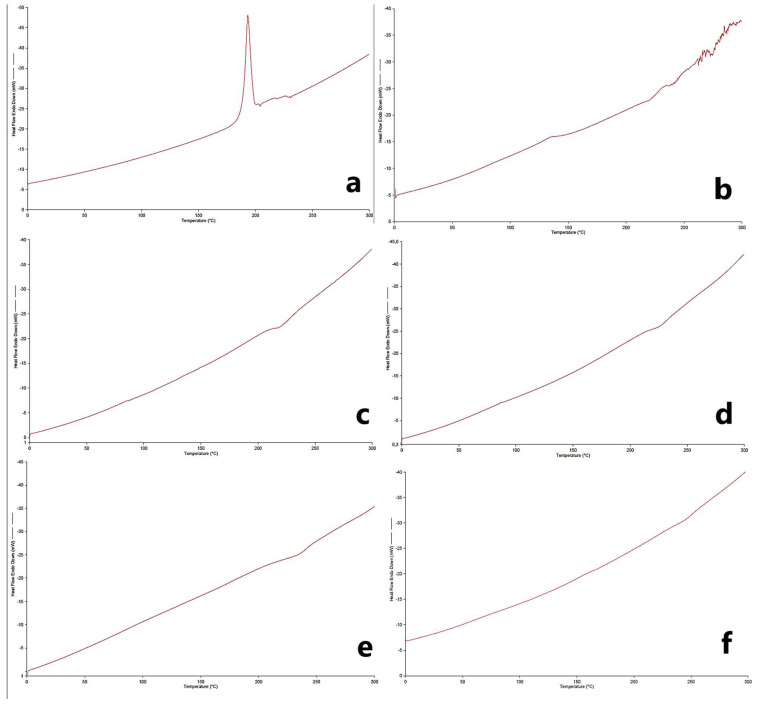
DSC thermograms of pure drugs, optimum formulations, and physical mixtures (**a**) PG, (**b**) MC, (**c**) F5, (**d**) F8, (**e**) F13, and (**f**) physical mixture.

The interactions of PG and MC with excipients were studied to elucidate the thermal properties of the drug to predict any possible physicochemical interactions that might affect the drug release rate or drug release mechanism from polymeric film formulations. The DSC thermograms obtained are given in Figure 7. When the thermograms are examined, a melting peak around 195–200 °C is observed for PG in accordance with the literature (Figure 7a) [36,38]. The characteristic melting endotherm for MC was observed at around 270–280 °C, consistent with the literature (Figure 7b). Characteristic endothermic peaks in the active substances were not observed in the physical mixture and formulations since it suggests the possibility of molecular dispersion by complete amorphization [34].

### 2.17. Surface and Structural Morphology

**Figure 8:**
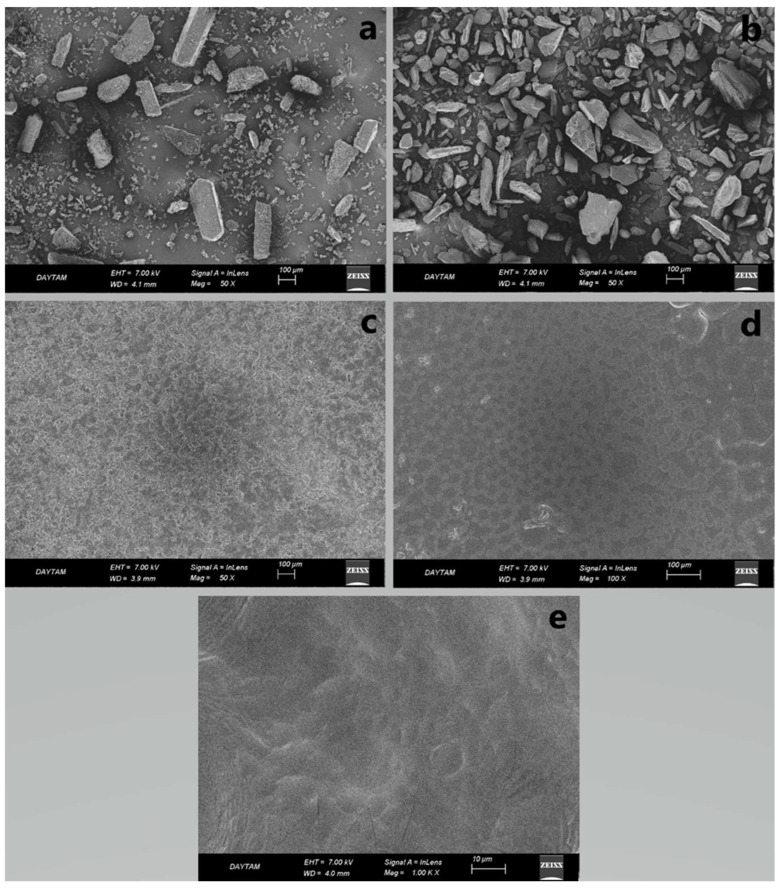
SEM images of pure drugs and selected formulations (**a**) PG, (**b**) MC, (**c**) F5, (**d**) F8, and (**e**) F13.

SEM imaging was performed to show the surface morphology of the films (Figure 8). The OTFs showed an irregular but homogeneous surface due to the low extrusion temperature. The surfaces of the prepared PG- and MC-containing hydrogel-based OTFs F5 and F8 were rough (Figure 8c,d). The F13 formulation was smooth. When we look at the literature, it is seen that there are similar film morphologies in similar studies [25]. However, the roughness/smoothness of an OTF does not have any effect on the drug release properties of the film.

### 2.18. Stability of OTFs

The stability of the prepared formulations was evaluated in a desiccator with 60% relative humidity at 25 °C and +4 °C for 12 months. At the end of the 12th month of measurement, the moisture absorption capacity, foldability, percent elongation, thickness, transparency, weight variability, % swelling degrees, content uniformity, and duration of formulations were examined (Table 9). The data obtained and the test/analysis results performed when the OTFs were freshly prepared were compared, and it was seen that there was no statistically significant difference (*p* < 0.05).

### 2.19. MTT Cell-Viability Assay

MTT analysis was used to assess cell viability. Cell viability was determined by assuming 100% of the data obtained for the negative control group and comparing other data accordingly [39,40,41].

**Figure 9:**
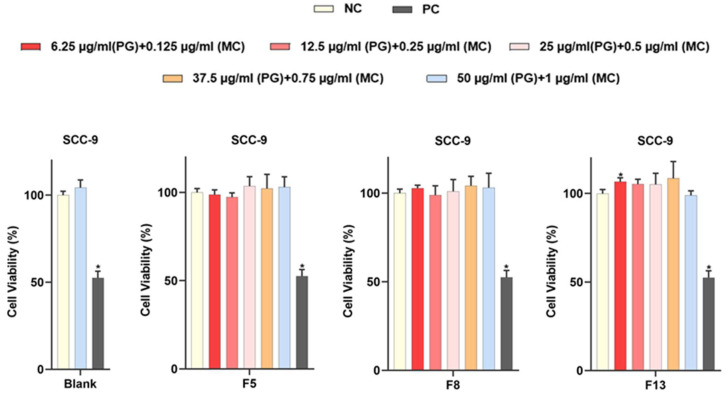
Cell viability test results of Blank, F5, F8, and F13. Negative control (NC) cells were grown to contain medium only. Positive control (PC) cells were treated with 10% DMSO. Statistical significance is shown as * (*p* < 0.05) compared to the NC.

As a result of various test and analysis studies, formulations with optimum properties were selected (F5, F8, and F13) and cell culture studies were carried out. The results obtained are given in Figure 9. When Figure 9 was examined, it was observed that cell viability was close to 100% at all doses of blank OTFs and PG- and MC-loaded OTFs.

MTT studies of OTFs in the literature were examined, and it was observed that the cytotoxicity test is performed in shorter periods (such as 0.5, 2, or 3 h). In our study, it was performed for 2 h in this aspect [42,43]. As a result, it was determined that hydrogel-based OTFs containing MC and PG did not cause toxicity even at high doses. We observed that 50 µg/mL PG dose and 1.0 µg/mL MC dose did not cause toxicity, which we tried as the highest dose for all our formulations. In the literature, Salat et al. reported that 100 µg/mL PG dose on HepG2 and 3T3-L1 cell lines did not have a negative effect on cell viability even after 24 h, and it maintained 100% cell viability [44]. Baldewig et al. reported that 10 µg/mL and 100 µg/mL PG doses did not cause any change in cell viability at the end of 2 h in their cytotoxicity study on the PC12 neuronal cell line and that the cells maintained 100% viability [42].

The characteristic abnormality in NP is a lesion or dysfunction of the sensory pathway with hyperexcitability of the neural region. It causes depression, fatigue, anxiety, sleep disturbances, and general physical function decline, accompanied by long-term pain. In peripheral neuropathy, various etiologies vary depending on the location of the nerve damage. Peripheral neuropathy is very common in type 2 diabetes mellitus (T2DM), a disease called diabetic peripheral neuropathy (DPN), affecting about half of patients with diabetes. Besides pain, another important clinical manifestation of DPN is insensitivity, which increases the risk of burning, injury, and foot ulcers [14].

PG and MC together relieve the symptoms of peripheral neuropathy. In a study, a fixed-dose combination of PG and MC (PG 75 mg and MC 750 μg) was administered to 1327 patients for 4 weeks in 300 orthopedic clinics. At the end of 4 weeks, the study was concluded with 80% doctor satisfaction and 88.11% excellent patient response to treatment. Tolerance to the drug was expressed as good to excellent in 81.48% of patients. In addition, the side effects were mild. Thus, it has been shown that the combination of fixed-dose PG and MC in NP offers an effective and good treatment with minimal side effects [45].

Water is the external liquid component of hydrogels [46]. Hydrogels are semi-solid systems in which a liquid phase is immobilized by a three-dimensional (3D) network of self-assembled, interlocking polymer/gel-forming agents. Research on these systems has gained momentum in the last few years [47]. Hydrogels are 3D polymer networks that can swell in aqueous solutions. Mucoadhesive and bioadhesive properties that increase drug resistance time and complete tissue permeability make hydrogels excellent drug delivery systems. Hydrogels have self-healing abilities even if their structure is damaged [48]. Hydrogels are used in a wide variety of biomedical and pharmaceutical applications due to their high water content and, consequently, their excellent biocompatibility [49]. The potential of this technology lies in its ability to release drugs in a controlled manner. These systems have the potential to replace conventional drug formulations due to their biodegradability, water absorption, and low toxicity as biocompatible polymers and outstanding rheological properties [50].

One of the newest trends in hydrogel research is towards smart hydrogels, which exhibit reversible sol-gel transitions when exposed to external stimuli such as temperature, pH, magnetic field, and light, and have potential applications in site-specific protein/peptide delivery systems. Among these intelligent hydrogels, those that are pH sensitive have been extensively studied because the pH value is a significant environmental factor in biomedical systems and is very easily controlled both in vitro and in vivo. In the last few decades, polymers of natural origin, mainly polysaccharides, have been frequently used to fabricate smart hydrogels as vehicles for controlled drug delivery because they are inherently biodegradable, biocompatible, and convenient in chemical modification, with renewability. In most cases, drugs are enveloped in the hydrogel matrix by only simple physical forces, resulting in a diffusion-controlled and immediate release of charged drugs due to relatively weak intermolecular interactions. The fusion of natural polysaccharides and artificial polymers can address these issues and integrate each ingredient’s benefits [51].

The use of polysaccharide-based hydrogels as a drug delivery carrier in biomedical and pharmaceutical applications has contributed to solving relatively complex biocompatibility problems due to their non-toxicity, biodegradability and biocompatibility. For example, Yang et al. reported that they developed a new oral insulin delivery system using chitosan-based hydrogel microparticles as a carrier for insulin to increase the efficiency of insulin administration. In this way, approximately 90% of the insulin was successfully retained in the hydrogels in the gastric environment and was released slowly after transitioning to the intestinal conditions. Thus, it has been shown that insulin can be used orally with hydrogels, and that, moreover, hydrogels may be a suitable route for oral delivery of proteins and peptides as well [52]. Yuwei et al. reported that they developed an acid-fast and physiological pH-sensitive DNA hydrogel. They reported that they aimed to transport insulin, especially orally, by encapsulating it in a DNA hydrogel in an acidic environment, and reported successful results in diabetic mice. They reported that DNA hydrogel could be potential carriers for oral drug delivery [53].

The existence of multi-alternative biocompatible polymers and the variability in production technologies have made it possible to develop a wide variety of thin films. Increasingly, thin films are gaining popularity and acceptance in the pharmaceutical industry as a new drug carrier dosage form. Much effort has been devoted to formulating polymeric thin films for application generally to the buccal, sublingual, ocular, and skin. Among these applications, the use of thin films for drug delivery from the sublingual or buccal mucosa has attracted great interest in recent years. Many pharmaceutical companies are impressed by the attractive properties of thin films, and, as a result, they continue to develop thin films technologies and still obtain patents for these formulations [9].

The oral route availability of many drugs is inadequate due to gastric pH, enzymes, and extensive first-pass effects through the liver. Drugs like this have traditionally been administered parenterally and have exhibited low patient compliance. This has paved the way for the pharmaceutical industry to develop alternative systems for the transport of drugs by developing orally dispersible/soluble thin films. When an OTF is placed on the tip or base of the tongue, it is instantly wet with saliva. As a result, it disperses and/or dissolves to liberate the drug for local and/or systemic absorption. The permeability of the sublingual and buccal mucosa is high due to the thin membrane structure and a large number of blood vessels. Due to this rapid blood circulation, there is very rapid bioavailability. Furthermore, oral films are beneficial for uncooperative patients, as they are given once in the mouth and are very difficult to remove. In addition, variables such as color and taste can be easily manipulated according to the preferences of the consumer/patient.

Thin-film dosage form drugs are taking a high market share today due to their ease of administration and high efficiency. While the OTF market for pharmaceutical products was 500 million dollars in 2007, it had reached 2 billion dollars by 2010. It is expected that the thin-film drug production market in the world pharmaceutical market will increase from 7 billion dollars in 2015 to over 15 billion dollars by the end of 2024. At this point, in many prescription and non-prescription product groups, especially in cough, cold, sore throat, erectile dysfunction disorders, allergic reactions, asthma, gastrointestinal disorders, pains (especially pain originating in the central nervous system), snoring complaints, sleep problems, multivitamin combinations, etc., their uses exist and continue to increase [54].

## 3. Conclusions

In this study, hydrogel-based OTFs containing PG and MC and which dissolve rapidly in the mouth in less than 30 s have been successfully developed. These thin film formulations provide an immediate release. They may offer an alternative and ease of use as new drug delivery vehicles, especially for NP patients including dysphagic patients, geriatric, pediatric, bedridden, and mentally challenged patients, in the future. Toxicity tests performed on the tongue epithelial cell line have proven these thin film formulations’ reliability. It is planned to support the obtained data in this work with further in vivo studies and detailed tests.

## 4. Materials and Methods

### 4.1. Material

PVP-CL, PVP-K25, HPMC K100, PVP-K30, and HPMC E-15 were obtained from Santa Farma İlaç Sanayii (İstanbul, Turkey). Pregabalin, croscarmellose sodium, and sodium starch glycolate were obtained from İlko İlaç (Ankara, Turkey). Methylcobalamin and pullulan were purchased from TCI (Tokyo, Japan). Sodium alginate was purchased from Alfa Aesar^®^ (Kandel, Germany). Citric acid was purchased from Honeywell–Fluka (Muskegon, MI, USA). Pectin was purchased from CPKelco (Lille Skensved, Denmark). Propylene glycol, D-sorbitol, and gelatin were purchsed from Acros Organics (Geel, Belgium). Mannitol was purchased from Merck^®^ (Darmstadt, Germany). Glycerine was purchased from CDH (Delhi, India). Aspartame was purchased from ABCR (Karlsruhe, Germany). Xanthan gum, sodium saccharin, sodium lauryl sulphate (SLS), NaCMC, Poloxomer 407 were purchased from Sigma–Aldrich^®^ (St. Louis, MO, USA). Vanillin, PEG 400, and PEG 4000 were purchased from Sigma-Aldrich^®^ (Darmstadt, Germany). Human Tongue Squamous Cell Carcinoma (SCC-9) was purchased from ATCC (Manassas, VA, USA). Gibco™ DMEM/F12 and Gibco™ FBS used for cell culture studies were purchased from ThermoFisher Scientific (Waltham, MA, USA). Penicillin/streptomycin, hydrocortizone, L-glutamine, PBS, trypsin/EDTA, and Cell Viability Detection Kit 8 (CVDK-8) were purchased from Ecotech Biotechnology (Erzurum, Turkey). Triton™ X-100 were purchased from Sigma–Aldrich^®^ (Darmstadt, Germany). Ultrapure water (Direct-Q^®^ 3 UV Millipore, Merck^®^, Darmstadt, Germany) was used in all formulations (18.2 MΩ·cm, TOC ≤ 4 ppb). Other chemicals used were of analytical or pharmaceutical grade. Figures were created using GraphPad Prism 9.0 (GraphPad Software, Inc., La Jolla, CA, USA).

### 4.2. Development and Validation of Pregabalin and Methylcobalamin Simultaneous Assay Method

PG and MC were developed and validated by modifying the quantitative determination methods in combined preparations in the literature. For this purpose, an HPLC (Agilent-1260, Waldbronn, Germany) device was used. During the study, various mobile phases and component ratios, flow times and amounts, and column and column temperatures were tried [55,56].

### 4.3. Preparation of Standard Solutions

Stock solutions were prepared by dissolving 5 mg of PG and 1 mg of MC, weighed accurately, separately in the appropriate pH 6.8 phosphate buffer. Standard solutions were prepared by making appropriate dilutions from these stock solutions and reading against the wavelengths at which they gave maximum absorbance in HPLC. The peak areas were plotted, and a calibration line and calibration equation to be used in all subsequent studies were obtained (*n* = 3).

### 4.4. Method Validation

Analytical method validation is defined as method validation. It is a mandatory requirement that the chosen method will yield reproducible and reliable results suitable for the objectives to be achieved. According to the ICH Q2 (R1) guideline, the developed method has been validated for linearity, accuracy, precision, the limit of detection (LOD), the limit of quantification (LOQ), and selectivity (specificity) [17,55,56].

#### 4.4.1. Linearity

The linearity of an analytical method is its ability to obtain test results within a certain range that is directly proportional to the concentration of the analyte in the sample. The linearity of the method was validated by determining the remaining concentrations within the calibration line.

#### 4.4.2. Accuracy and Precision

Accuracy can be defined as the degree of closeness between the accepted actual value and the analyzed reference value. Precision means the closeness of the measurements of analyte amounts in samples prepared from the same homogeneous sample under prescribed conditions. In order to determine the accuracy and precision of the method, repeated experiments were carried out within and between days. For this purpose, three points on the calibration curve were determined; analyses were made for three consecutive days, intraday and interday. The results were statistically compared with the values that should be. Accuracy is expressed in % Relative Error (RE%) and Precision in % Relative Standard Deviation (RSD%).

#### 4.4.3. LOD

The lowest amount of analyte that can be detected in the sample but not counted as an accurate and precise value is called the detection limit. It has been determined experimentally.

#### 4.4.4. LOQ

The limit of quantitation is the lowest amount of analyte that can be quantified with a certain level of accuracy and precision. It has been determined experimentally.

#### 4.4.5. Selectivity (Specificity)

The specificity of the method was determined by analyzing and detecting PG and MC in the presence of all excipients that make up the formulations. Thus, it was determined whether the method was specific to the analyzed active substances.

#### 4.4.6. Stability of Active Substances

The stability of the active substances in the stock solution is important in the assay process. For this purpose, the stability of PG and MC was evaluated in stock solution and at room temperature conditions, provided that they were kept for 72 h.

### 4.5. Preparation of OTFs

**Figure 10:**
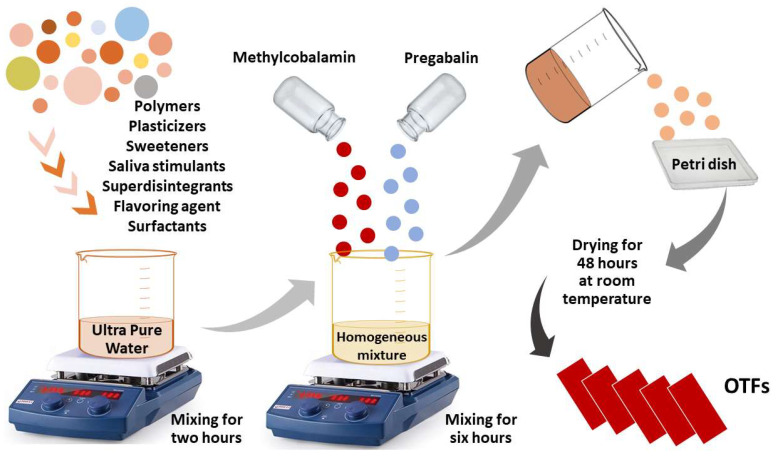
Preparation of OTFs.

OTFs were prepared using the Solvent Pouring Method [5,20,57]. Water-soluble film-forming polymers (such as 40–50%, pullulan, pectin, sodium alginate, gelatin, HPMC, NaCMC, PVP-K25, PVP-K30) and plasticizers (0–20%, glycerin, PEG-400, PEG-4000, propylene glycol, such as D-sorbitol) were dissolved in a beaker containing ultrapure water. The prepared solution was mixed on a heated magnetic stirrer by applying the appropriate time (such as 30 min, 1 h, 2 h) and temperature (such as 30 °C, 40 °C, 50 °C). It was waited for a while to remove all air bubbles formed in the solution. Afterward, sweeteners (3–6%, mannitol, aspartame, sodium saccharin), saliva stimulant (2–6%, citric acid), superdisintegrants (0–8%, sodium starch glycolate, crospovidone, croscarmellose sodium, xanthan gum, etc.), flavoring agent (vanillin) and surfactants (such as SLS, Poloxamer 407) were added sequentially into the formulation and dissolved. Finally, 25 mg of PG and 0.5 mg of MC were added to the formulation. It was mixed at 750 rpm for 6 h to ensure a homogeneous mixture and a temperature equal to room temperature. These viscous solutions, which contained PG and MC in equal doses and equal volumes, were poured into plastic square petri dishes to form a film (the internal volume and the dose to be taken having been calculated), and the formed films were dried at room temperature conditions for 48 h. These elastic films obtained after drying were removed from the petri dishes and divided into equal parts so that each film contained an equal amount of PG-MC. All prepared formulations were stored at room temperature in the dark and in a non-humid environment. The preparation of PG-MC-free (blank) OTFs for use in analysis, measurement, and evaluation was also performed in the same way as described above, without the addition of PG-MC. All series were studied in at least three repetitions. In this context, many formulations have been developed, but 24 formulations have been selected as a result of these preformulation studies. Formulation codes and components are given in Table 10 below. The preparation of OTFs is schematically given in Figure 10.

### 4.6. Organoleptic Evaluation of OTFs

Visual properties such as the homogeneity, color, texture, and thickness of the OTFs containing PG-MC were determined. They were was also evaluated in terms of taste and flavor properties.

### 4.7. Moisture Absorption Capacity

This test was performed to check the physical stability and integrity of the films in high-humidity conditions. After the formulations were weighed individually, they were placed in desiccators containing desiccants, and it was observed how much moisture they lost in their structure over three days. Afterward, the films were weighed, and their % moisture absorption capacity was calculated with the following formula (*n* = 3) [58,59]:

% *Moisture Content* = 100 × (*OTF Final Weight* − *OTF Initial Weight*)/*OTF Initial Weight*

### 4.8. Tensile Strength (Elongation)

When a pulling force is applied, the tension increases. This tension continues until the integrity of the film form is destroyed. The percent elongation can be determined by measuring the final length of the film before its integrity is broken. This ratio increases as the plasticizer content increases [1]. The films must maintain this flexibility in terms of structural integrity and stability. The elongation percentages of the prepared OTF formulations were determined with a tensile strength tester (Shimadzu AGS-X). The thin films were attached between the upper pulling arm and the lower pulling arm of the instrument. The percent elongation was calculated by measuring the lengths of the OTFs before and at the beginning by pulling these arms backwards mutually at one-second intervals. In addition, the tensile force for each OTF was read and recorded at the exact moment of rupture [60,61,62,63]. This test was performed in a minimum of three replicates for each formulation. The films’ tensile strength was calculated with the following formula:

% *Elongation* = 100 × (*increase in OTF length*/*OTF initial length*)

### 4.9. Weight Variability

Films of 1 × 1 cm^2^ size were cut from each OTF formulation and weighed individually on a precision balance to calculate weight variability (*n* = 6) [64].

### 4.10. Thickness

Thickness measurement is necessary because it is directly related to the amount of drug in the film. However, an appropriate thickness is required for the comfortable application of the films. For this purpose, at least five films from each OTF formulation were measured from five different points with a manual caliper. Results are given as arithmetic mean and standard deviation (Ⴟ and SD) [8].

### 4.11. Folding Endurance (Flexibility)

The folding endurance of OTFs is determined by repeatedly folding a film at an angle of 180° onto the same side until it breaks. The final number of folds of the film before breaking is noted. Films that exhibit fold strength of 300 times or more are considered to have excellent flexibility [8,64]. For this purpose, each OTF formulation was subjected to this test (*n* = 3). The results are given as Ⴟ and SS.

### 4.12. Determination of pH Values

Determining the pH of OTFs is important in terms of their solubility/dispersibility in the oral cavity, taste characteristics, and rapid release of active substances in the mouth [8,63]. For this purpose, 5 mL of artificial salivary fluid (pH 6.8) was added to a beaker, and OTF samples containing full-dose active substances were placed and swelling was ensured. After swelling, pH values were determined using a pH meter (WTW, Inolab, Weilheim, Gemany).

### 4.13. Determination of Swelling Degrees

It is important to measure the swelling of the polymeric film or the water absorption capacity of OTFs and to obtain information about their resistance to water. The randomly selected OTFs were individually weighed and then placed in beakers filled with the appropriate amount of artificial salivary fluid (pH 6.8) for a specified period of time; this was carried out in a horizontal shaker water bath set at 37 °C (*n* = 6). Each OTF was weighed and measured at different time intervals until the increase in film weight reached a constant weight. The degree of swelling in % was calculated using the following equation [9]:

% *Swelling Degree* = 100 × (*OTF Final Weight* − *OTF Initial Weight*)/*OTF Initial Weight*

### 4.14. Disintegration Test

Disintegration time is defined as the corruption of the structural integrity of a film when it comes into contact with water or saliva. This is the time (seconds) that a film begins to break up or fall apart. Therefore, the thickness and weight of OTFs play an important role in determining the physical properties of the films [2].

Disintegration devices specified in the pharmacopeia are used to determine the disintegration times of OTFs. Normally, the disintegration time is a phenomenon that varies with the film composition/formulation and generally ranges from 5 to 30 s. There is no official guideline for determining the disintegration times of orally disintegrating films. The experiment was conducted by placing an OTF in 5 mL of artificial salivary fluid (pH 6.8) in a beaker in a horizontal shaker water bath set at 37 °C and a speed of 100 rpm. This experiment was run in six replicates for each OTF formulation. The time taken for the OTFs to completely dissolve/disintegrate was considered to be their disintegration time [21].

### 4.15. Transparency

The transparency of OTFs can be determined using a simple UV spectrophotometer. OTF formulation samples were cut into rectangles and placed inside the UV spectrophotometer cuvette, and measured at 600 nm wavelength. The following equation is used to interpret the transparency of OTFs [21]:

*Transparency* = *logT*600/*b* = −*€c*

*T*600 = Transmittance at 600 nm, *b* = Film thickness (mm), *c* = concentration.

Successful formulations (F5, F8, F13) as a result of pre-formulation studies were determined with organoleptic control, transparency, flexibility (foldability), percent elongation, moisture loss, disintegration time, pH values, and swelling degrees. Other tests and analyses below were conducted on these selected formulations.

### 4.16. Content Uniformity

OTFs were dissolved in 10 mL of artificial salivary fluid (pH 6.8) and passed through 0.45 µm membrane filters. The active ingredient content in each film was determined by the validated HPLC method. The BSS% should not be more than 6%. This experiment was performed in triplicate for each selected OTF formulation [8].

### 4.17. Dissolution Rate Test

In the literature, many studies have improvised the apparatus used for dissolution rate testing, while others have used Franz diffusion cells to test drug release from polymeric films. The biggest obstacle in the dissolution rate test is the placement of the film samples. In addition, various methods have been applied in the literature, in which the film is attached to the bottom of the glass chamber of the dissolution rate device or to the mixing apparatus using a double-sided adhesive tape [8]. In our study, OTFs were fixed to the bottom of the beaker using a weight. It was studied in beakers filled with 50 mL of artificial salivary fluid (pH 6.8) at 100 rpm in a horizontal shaker water bath set at 37 °C. The experiment was performed in triplicate for each OTF formulation group. Within certain times (30 s, 1 min, 2.5 min, 5 min, and 10 min), 2 mL samples were taken from each beaker, and then the same amount of fresh artificial salivary fluid (mean intraoral salivary fluid volume 0.52–2.14 mL) was added to each beaker to maintain sink conditions. The samples were passed through 0.45 µm membrane filters. The amounts of active substances in the samples taken in each time period were determined by the validated HPLC method [64].

### 4.18. Determination of Release Kinetics

The results were calculated using the Microsoft Excel program to determine which kinetic model and release mechanism the selected OTF formulations had after the dissolution rate test. It was determined by mathematical calculations and formulas that the formulations would be compatible with the Zero Order, First Order, Korsmeyer-Peppas, or Higuchi kinetic models [27].

### 4.19. FT-IR Analysis

In order to determine whether there were undesirable interactions between formulation components and active ingredients, infrared spectra were taken from pure PG, MC and powder samples of formulations with an FTIR (ATR) spectrometer (Bruker VERTEX 70v, Billerica, MA, USA) in the wavenumber range of 4000–400 cm^−1^.

### 4.20. XRD Analysis

X-ray diffraction analysis helps to determine the crystalline or amorphous nature of the active ingredients used in the films. For this purpose, an X-ray diffractometer (PANalytical Empyrean XRD, Almelo, The Netherlands) with 45 kV voltage and 40 mA current was used to analyze the PG, MC, and conformational sequences in the formulations chosen for this purpose [54].

### 4.21. DSC Analysis

DSC analysis is done to show the compatibility of the active ingredients with other excipients. The reference and sample are brought to the same temperature and the interactions in the sample due to heat change are examined [8]. Exactly weighed OTF samples were placed in an aluminum pan and analyzed under nitrogen atmosphere to show the compatibility of formulation components and active ingredients. Measurements were carried out at a temperature range of 0 °C to 300 °C and a heating rate of 10 °C/min.

### 4.22. Surface and Structural Morphology

Surface and structural morphology were examined using Scanning Electron Microscopy (SEM; Zeiss, Sigma 300, Jena, Germany). For analysis, a small piece of film was placed on the carbon tape and worked after it was covered with a thin layer (100 Å) of gold, which is an insulator [8]. The presence of pores, surface roughness, and homogeneity in the OTF formulations was determined.

### 4.23. Stability of OTFs

The stability of the prepared formulations was evaluated in a desiccator with 60% relative humidity at 25 °C and +4 °C for 12 months. For the measurements, at the end of the 12th month, the moisture absorption, flexibility, percent elongation, thickness, transparency, weight variability, folding strength, swelling degrees, active ingredient determination, and release studies of the formulations were compared with the freshly prepared formulation data. Each OTF sample in a light-protected package was placed in a closed desiccator. Measurements were performed in a minimum of three replicates for each formulation group and parameter. Whether the difference between the results was significant was evaluated statistically [65,66,67].

### 4.24. Cell Culture and MTT Cell-Viability Assay

Cell culture studies were carried out at the High Technology Research Center (YÜTAM) at Erzurum Technical University.

Human Tongue Squamous Cell Carcinoma (SCC-9) cells were cultured in DMEM F12 medium containing 10% FBS, 1% Penicillin/Streptomycin, 1% L-Glutamine, and 400 ng/mL Hydrocortisone. Cells were incubated in a humidified incubator at 37 °C and 5% CO_2_.

SCC-9 cells grown in cell culture flasks were removed from the adhered surface using Trypsin/EDTA. The total cell number was calculated by the trypan blue method. Cells were seeded into 96-well plates in triplicate, with 3 × 10^3^ cells per well. Each OTF (containing 25 mg of PG and 0.5 mg of MC) was dissolved in 5 mL of ultrapure water. Based on this stock solution, containing PG (at concentrations of 50, 37.5, 25.0, 12.5, and 6.25 µg/mL) and MC (at concentrations of 1.0, 0.75, 0.5, 0.25, and 0.125 µg/mL) samples were applied to all wells in triplicate with a total volume of 100 μL in each well. Positive control wells were treated with 10% DMSO. It was incubated for 24 h at 37 °C and 5% CO_2_ conditions. At the end of the incubation period, the medium in the wells was removed, and the medium containing 10% CVDK-8 solution was added to each well. Cells were incubated in the dark at 37 °C for up to 3 h in an incubator. Cell viability was determined by measuring optical density at 450 nm with an Epoch 2 Microplate Spectrophotometer (BioTek, Winooski, VT, USA). Changes in cell viability were calculated with reference to control groups [25,31,43]. The contents and quantities of formulations used for cell culture are given in Table 11.

### 4.25. Statistical Evaluations

All data were presented as the arithmetic mean ± standard deviation (Ⴟ ± SD). Statistical analysis was performed to determine whether there were any statistically significant differences between moisture sorption, percent elongation, thickness, transparency, weight variability, elasticity, degrees of swelling, and stability. The disintegration and dissolution rate of formulations was obtained with the one-way analysis of variance (ANOVA) by IBM SPSS Statistics 20. Cell culture analyses were performed by Student’s *t*-test. Differences were considered to be statistically significant at *p* < 0.05.

## Figures and Tables

**Table 1:** Chromatographic conditions.

Mobile Phase	0.05 M pH 3.5 Potassium Dihydrogen Orthophosphate Buffer (KH_2_PO_4_):ACN (92:8)
Column	ACE C_18_, 250 × 4.6 mm, 5 µm
Wavelength (λ)	205 nm
Column temperature	30 °C
Flow and Duration	1 mL/min
Injection Volume	20 μL

**Table 2:** Quantification method validation for intraday and interday accuracy and precision data (*n* = 3, Ⴟ ± SD).

For MC									
	Theoretical (µg/mL)	Detected (µg/mL)	RSD %	RE %		Theoretical (µg/mL)	Detected (µg/mL)	RSD %	RE %
	1.25	1.2 ± 0.51	1.31 ± 0.23	−1.24 ± 0.48		1.25	1.2 ± 0.33	1.77 ± 0.33	−0.81 ± 0.03
**Intraday**	12.5	12.6 ± 0.93	1.41 ± 0.54	1.06 ± 0.26	**Interday**	12.5	12.6 ± 0.76	1.17 ± 0.40	0.87 ± 0.18
	75	75.8 ± 1.15	0.67 ± 0.33	1.09 ± 0.39		75	75.1 ± 1.38	1.51 ± 0.27	0.13 ± 0.08
**For PG**									
	37.5	37.8 ± 0.90	2.05 ± 0.19	0.78 ± 0.63		37.5	38.5 ± 1.41	2.01 ± 0.16	2.72 ± 0.41
**Intraday**	150	148.4 ± 2.15	2.41 ± 0.26	−1.04 ± 0.51	**Interday**	150	148.4 ± 2.08	1.70 ± 0.34	−1.04 ± 0.30
	450	461.0 ± 4.28	0.37 ± 0.43	2.45 ± 0.18		450	459.4 ± 3.13	0.53 ± 0.26	2.08 ± 0.11

**Table 3:** PG and MC recovery % data after 72 h (Ⴟ ± SD).

Recovery %	
MC	PG
100.00 ± 2.03	101.39 ± 2.98
98.29 ± 2.68	103.83 ± 2.37
98.15 ± 0.84	98.91 ± 1.06

**Table 4:** Organoleptic evaluation of OTF formulations.

		Taste	Smell	Texture	Clarity	Thickness (µm)	Flexibility	Homogeneity
**Formulation Codes**	**F1**	✓	✓	Rough	Clear	30 ± 0.01	Fragile	—
	**F2**	—	✓	Rough	Opaque	30 ± 0.00	Fragile	—
	**F3**	✓	—	Rough	Semi Clear	30 ± 0.01	Fragile	—
	**F4**	—	✓	Smooth	Opaque	30 ± 0.01	Fragile	✓
	**F5**	✓	✓	Smooth	Opaque	30 ± 0.01	✓	✓
	**F6**	—	✓	Smooth	Semi Clear	30 ± 0.00	✓	✓
	**F7**	—	✓	Rough	Opaque	30 ± 0.00	Fragile	—
	**F8**	✓	✓	Smooth	Opaque	30 ± 0.00	✓	✓
	**F9**	—	✓	Smooth	Opaque	30 ± 0.01	Fragile	✓
	**F10**	—	✓	Smooth	Opaque	30 ± 0.01	Fragile	✓
	**F11**	—	✓	Smooth	Semi Clear	30 ± 0.00	✓	—
	**F12**	—	—	Smooth	Clear	30 ± 0.01	✓	✓
	**F13**	✓	✓	Smooth	Opaque	30 ± 0.00	✓	✓
	**F14**	—	✓	Smooth	Semi Clear	30 ± 0.00	✓	✓
	**F15**	—	—	Smooth	Opaque	30 ± 0.01	✓	✓
	**F16**	✓	—	Smooth	Opaque	30 ± 0.01	✓	✓
	**F17**	✓	—	Smooth	Semi Clear	30 ± 0.00	✓	—
	**F18**	—	✓	Rough	Opaque	30 ± 0.01	Fragile	—
	**F19**	—	—	Rough	Opaque	30 ± 0.00	Fragile	—
	**F20**	—	✓	Rough	Opaque	30 ± 0.00	Fragile	✓
	**F21**	—	—	Rough	Semi Clear	30 ± 0.01	Fragile	—
	**F22**	—	—	Rough	Semi Clear	30 ± 0.01	Fragile	✓
	**F23**	—	✓	Rough	Opaque	30 ± 0.01	Fragile	—
	**F24**	—	✓	Rough	Semi Clear	30 ± 0.01	Fragile	—

**Table 5:** Tests and analyses on OTF formulations (Ⴟ ± SD).

		Moisture Uptake Capacity	Tensile Strength	Weight Variability	Folding Endurance	pH	Swelling Degree	Disintegration Time (s)	Transparency
**Formulation Codes**	**F1**	1.0 ± 0.89	0.0 ± 0.0	0.02 ± 0.0	5 ± 1.5	6.02 ± 0.5	576 ± 45	477 ± 13	206 ± 8.6
	**F2**	0.88 ± 0.39	0.0 ± 0.0	0.02 ± 0.0	10 ± 2.0	6.05 ± 0.2	810 ± 35	121 ± 8.0	70 ± 3.1
	**F3**	2.4 ± 0.35	0.0 ± 0.0	0.03 ± 0.0	22 ± 4.0	6.01 ± 0.7	643 ± 28	92 ± 5.0	129 ± 5.6
	**F4**	0.8 ± 0.11	6.0 ± 0.3	0.03 ± 0.0	150 ± 8.0	5.98 ± 0.3	269 ± 12	74 ± 6.0	97 ± 4.3
	**F5**	1.2 ± 0.25	2.7 ± 0.2	0.03 ± 0.0	200 ± 8.5	5.50 ± 0.2	203 ± 11	28 ± 2.0	63 ± 2.1
	**F6**	1.5 ± 0.24	1.6 ± 0.01	0.03 ± 0.0	200 ± 12	5.55 ± 0.4	316 ± 10	62 ± 6.0	131 ± 5.1
	**F7**	0.7 ± 0.05	0.0 ± 0.0	0.03 ± 0.0	10 ± 1.5	5.45 ± 0.5	270 ± 8.0	27 ± 4.0	85 ± 3.2
	**F8**	0.7 ± 0.13	10.6 ± 1.5	0.03 ± 0.0	215 ± 11	6.05 ± 0.1	111 ± 6.0	17 ± 2.0	50 ± 3.0
	**F9**	0.8 ± 0.39	0.0 ± 0.0	0.03 ± 0.0	50 ± 6.5	5.78 ± 0.5	259 ± 10	51 ± 3.0	63 ± 4.8
	**F10**	0.7 ± 0.13	0.0 ± 0.0	0.03 ± 0.0	42 ± 3.5	5.11 ± 0.2	408 ± 12	67 ± 5.0	66 ± 3.4
	**F11**	1.4 ± 0.04	40.6 ± 2.4	0.02 ± 0.0	300 ± 17	5.45 ± 0.5	176 ± 7.0	82 ± 6.0	158 ± 6.7
	**F12**	1.6 ± 0.40	25.4 ± 1.2	0.03 ± 0.0	300 ± 14	5.25 ± 0.4	292 ± 17	66 ± 4.0	179 ± 8.1
	**F13**	0.47 ± 0.09	2.89 ± 0.48	0.03 ± 0.0	110 ± 4.5	6.55 ± 0.1	169 ± 11	16 ± 2.0	59 ± 2.9
	**F14**	1.1 ± 0.15	21.3 ± 4.3	0.02 ± 0.0	115 ± 8.5	6.30 ± 0.1	358 ± 10	246 ± 15	95 ± 2.7
	**F15**	1.7 ± 0.17	2.7 ± 1.0	0.03 ± 0.0	300 ± 13	5.40 ± 0.2	180 ± 16	91 ± 3.0	72 ± 4.6
	**F16**	1.0 ± 0.15	4.0 ± 0.02	0.04 ± 0.0	300 ± 9.0	5.50 ± 0.2	215 ± 15	48 ± 3.0	83 ± 3.5
	**F17**	1.5 ± 0.36	14.1 ± 1.1	0.03 ± 0.0	240 ± 11	5.60 ± 0.1	347 ± 15	129 ± 9.0	169 ± 8.0
	**F18**	1.5 ± 0.25	0.0 ± 0.0	0.03 ± 0.0	65 ± 9.0	6.02 ± 0.3	238 ± 8.0	27 ± 3.0	60 ± 2.7
	**F19**	1.6 ± 0.10	9.0 ± 0.44	0.03 ± 0.0	150 ± 7.5	6.15 ± 0.1	434 ± 11	18 ± 2.0	49 ± 2.1
	**F20**	0.4 ± 0.04	3.8 ± 1.42	0.03 ± 0.0	100 ± 11	6.31 ± 0.4	376 ± 7.0	16 ± 2.0	57 ± 4.0
	**F21**	0.3 ± 0.09	1.0 ± 0.8	0.02 ± 0.0	75 ± 7.5	5.31 ± 0.2	189 ± 13	122 ± 2.7	131 ± 6.3
	**F22**	1.1 ± 0.24	0.0 ± 0.0	0.03 ± 0.0	28 ± 8.5	6.32 ± 0.3	211 ± 36	131 ± 4.1	146 ± 0.7
	**F23**	0.8 ± 0.37	6.5 ± 0.3	0.03 ± 0.0	85 ± 12	5.40 ± 1.5	382 ± 14	68 ± 3.2	82 ± 3.3
	**F24**	1.3 ± 0.56	2.0 ± 0.0	0.03 ± 0.0	53 ± 3.0	5.38 ± 0.4	319 ± 42	97 ± 4.7	101 ± 5.0

**Table 6:** Content uniformity results of OTFs (*n* = 3, Ⴟ ± SD).

**For** **PG**	**Formulation Codes**	**Amount (mg)**	**For** **MC**	**Formulation Codes**	**Amount (mg)**
	**F5**	24.897 ± 0.518		**F5**	0.514 ± 0.013
	**F8**	25.347 ± 0.196		**F8**	0.518 ± 0.015
	**F13**	25.127 ± 0.583		**F13**	0.496 ± 0.014

**Table 7:** Dissolution rate results of OTFs (*n* = 3, Ⴟ ± SD).

**For** **PG**	**Formulation** **Codes**	**Time** **(min)**	**Cumulative** **Released %**	**For** **MC**	**Formulation** **Codes**	**Time** **(min)**	**Cumulative** **Released %**
	**F5**	0.5	9.96 ± 0.81		**F5**	0.5	16.81 ± 0.45
		1	33.83 ± 1.51			1	52.69 ± 0.85
		2.5	75.70 ± 0.66			2.5	74.36 ± 1.15
		5	101.16 ± 1.9			5	91.83 ± 1.49
		10	100.11 ± 0.4			10	100.09 ± 0.9
	**F8**	0.5	12.78 ± 0.23		**F8**	0.5	34.93 ± 0.33
		1	34.40 ± 0.39			1	65.68 ± 0.76
		2.5	72.89 ± 1.13			2.5	92.81 ± 0.88
		5	100.60 ± 0.9			5	104.95 ± 1.2
		10	100.34 ± 0.7			10	104.40 ± 0.2
	**F13**	0.5	4.73 ± 0.12		**F13**	0.5	15.63 ± 0.69
		1	31.20 ± 0.55			1	67.17 ± 1.06
		2.5	73.78 ± 0.67			2.5	94.38 ± 0.29
		5	104.21 ± 0.9			5	104.47 ± 0.8
		10	104.07 ± 0.9			10	105.15 ± 1.1

**Table 8:** Results of release kinetics of OTFs.

Formulation Codes	Drug	Zero Order	First Order	Higuchi	Korsmeyer-Peppas	
		R^2^	R^2^	R^2^	R^2^	*n*
**F5**	PG	0.66	0.52	0.82	0.85	0.762
	MC	0.66	0.49	0.80	0.81	0.534
**F8**	PG	0.69	0.55	0.84	0.88	0.693
	MC	0.53	0.47	0.69	0.80	0.537
**F13**	PG	0.68	0.48	0.81	0.84	0.987
	MC	0.46	0.36	0.62	0.68	0.550

**Table 9:** Long-term stability test results of selected formulations (Ⴟ ± SD).

Test/Analysis	+4 °C			+25 °C		
	F5	F8	F13	F5	F8	F13
**Moisture Uptake Capacity**	1.22 ± 0.11	0.68 ± 0.23	0.48 ± 0.09	1.31 ± 0.41	0.73 ± 0.60	0.55 ± 0.13
**Foldability**	195 ± 5.50	205 ± 9.50	110 ± 6.50	190 ± 7.00	200 ± 5.50	100 ± 3.50
**Elongation Percent**	2.51 ± 0.14	9.67 ± 1.13	2.66 ± 0.59	2.43 ± 0.18	9.15 ± 1.81	2.51 ± 0.66
**Thickness (µm)**	30 ± 0.01	30 ± 0.01	30 ± 0.01	30 ± 0.01	30 ± 0.01	30 ± 0.01
**Transparency**	65 ± 3.20	47 ± 1.80	61 ± 2.50	64 ± 2.00	49 ± 1.70	57 ± 1.90
**Weight Variability**	0.03 ± 0.00	0.03 ± 0.00	0.03 ± 0.00	0.03 ± 0.00	0.03 ± 0.00	0.03 ± 0.00
**Swelling Degree**	197 ± 9.89	107 ± 7.45	159 ± 9.12	207 ± 3.32	109 ± 12.48	171 ± 6.18
**Content Uniformity for PG (mg)**	25.118 ± 0.32	24.883 ± 0.29	25.212 ± 0.42	25.325 ± 0.61	25.089 ± 0.12	24.913 ± 0.35
**Content Uniformity for MC (mg)**	0.497 ± 0.02	0.499 ± 0.01	0.511 ± 0.02	0.505 ± 0.01	0.488 ± 0.02	0.520 ± 0.03
**Disintegration Time (s)**	29 ± 2.87	18 ± 1.93	17 ± 1.46	30 ± 2.52	18 ± 2.18	17 ± 1.77

**Table 10:** OTF formulations and components (mg) containing PG-MC.

	Formulation Codes																							
	F1	F2	F3	F4	F5	F6	F7	F8	F9	F10	F11	F12	F13	F14	F15	F16	F17	F18	F19	F20	F21	F22	F23	F24
**Polymers**																								
**Pullulan**	-	-	-	-	-	50	50	50	50	50	50	50	25	25	25	25	20	50	-	-	-	-	-	-
**NaCMC**	50	-	-	-	-	-	-	-	-	-	-	-	-	25	-	-	10	-	50	-	-	-	-	-
**Pectin**	-	-	25	25	-	-	-	-	-	-	-	-	-	-	25	12.5	10	-	-	50	-	-	-	-
**Sodium Alginate**	-	50	25	25	50	-	-	-	-	-	-	-	25	-	-	12.5	10	-	-	-	-	-	-	-
**PVP K-25**	-	-	-	-	-	-	-	-	-	-	-	-	-	-	-	-	-	-	-	-	50	-	-	-
**PVP K-30**	-	-	-	-	-	-	-	-	-	-	-	-	-	-	-	-	-	-	-	-	-	50	-	-
**HPMC E15**	-	-	-	-	-	-	-	-	-	-	-	-	-	-	-	-	-	-	-	-	-	-	50	-
**HPMC K100**	-	-	-	-	-	-	-	-	-	-	-	-	-	-	-	-	-	-	-	-	-	-	-	50
**Plasticizers**																								
**Glycerin**	-	-	-	25	25	25	-	25	-	-	25	25	25	25	25	25	25	-	-	-	-	-	-	-
**Propylene Glycol**	25	25	25	-	-	-	25	-	25	25	-	-	-	-	-	-	-	25	-	-	-	-	-	-
**PEG 400**	-	-	-	-	-	-	-	-	-	-	-	-	-	-	-	-	-	-	25	-	-	-	25	-
**PEG 4000**	-	-	-	-	-	-	-	-	-	-	-	-	-	-	-	-	-	-	-	25	-	-	-	25
**D-Sorbitol**	-	-	-	-	-	-	-	-	-	-	-	-	-	-	-	-	-	-	-	-	25	-	-	-
**PVA (low MW)**	-	-	-	-	-	-	-	-	-	-	-	-	-	-	-	-	-	-	-	-	-	25	-	-
**Saliva Stimulant**																								
**Citric Acid**	5	5	5	5	5	5	5	5	5	5	5	5	5	5	5	5	5	5	5	5	5	5	5	5
**Sweeteners**																								
**Sodium Saccharin**	10	10	10	10	10	10	10	10	10	10	10	10	10	10	10	10	10	-	-	-	-	-	-	-
**Mannitol**	10	10	10	10	10	10	10	10	10	10	10	10	10	10	10	10	10	10	10	10	10	10	10	10
**Aspartame**	-	-	-	-	-	-	-	-	-	-	-	-	-	-	-	-	-	10	10	10	10	10	10	10
**Superdisintegrants**																								
**NaStarch Glycolate**	-	-	-	-	-	15	-		15	-	15	-	-	-	-	-	-	-	-	-	-	-	15	15
**Xanthan Gum**	15	15	15	-	-	-	-	-	-	15	-	15	-	-	-	-	-	-	-	-	15	-	-	-
**NaCroscarmellose**	-	-	-	15	15	-	15	15	-	-	-	-	15	15	15	15	15	-	15	15	-	-	-	-
**PVP-CL**	-	-	-	-	-	-	-	-	-	-	-	-	-	-	-	-	-	15	-	-	-	15	-	-
**Flavoring Agent**																								
**Vanillin**	20	20	20	20	20	20	20	20	20	20	20	20	20	20	20	20	20	20	20	20	20	20	20	20
**Surfactants**																								
**SLS**	5	5	5	5	5	5	5	5	5	5	5	5	5	5	5	5	5	5	5	5	5	5	5	5
**Poloxamer 407**	5	5	5	5	5	5	5	5	5	5	5	5	5	5	5	5	5	5	5	5	5	5	5	5
**Ultrapure Water**																								
	qs	qs	qs	qs	qs	qs	qs	qs	qs	qs	qs	qs	qs	qs	qs	qs	Qs	qs	qs	qs	qs	qs	qs	qs

**Table 11:** Contents and quantities of formulations used for cell culture.

Formulation Codes	Contents	Concentration	Storage Condition
Blank	-	-	+4 °C
F5	PG + MC	5 mg/mL + 0.1 mg/mL	+4 °C
F8	PG + MC	5 mg/mL + 0.1 mg/mL	+4 °C
F13	PG + MC	5 mg/mL + 0.1 mg/mL	+4 °C

## Data Availability

Not applicable.
